# Snow microbiome functional analyses reveal novel aspects of microbial metabolism of complex organic compounds

**DOI:** 10.1002/mbo3.1100

**Published:** 2020-08-06

**Authors:** Chengsheng Zhu, Maximilian Miller, Nicholas Lusskin, Benoît Bergk Pinto, Lorrie Maccario, Max Häggblom, Timothy Vogel, Catherine Larose, Yana Bromberg

**Affiliations:** ^1^ Department of Biochemistry and Microbiology Rutgers University New Brunswick NJ USA; ^2^ Environmental Microbial Genomics Laboratoire Ampere Ecole Centrale de Lyon CNRS UMR 5005 Université de Lyon Ecully France; ^3^ Section of Microbiology Copenhagen University Copenhagen Ø Denmark; ^4^ Department of Genetics Human Genetics Institute Rutgers University Piscataway NJ USA

**Keywords:** metagenome, metatranscriptome, mi‐faser, snow microbiome

## Abstract

Microbes active in extreme cold are not as well explored as those of other extreme environments. Studies have revealed a substantial microbial diversity and identified cold‐specific microbiome molecular functions. We analyzed the metagenomes and metatranscriptomes of 20 snow samples collected in early and late spring in Svalbard, Norway using mi‐faser, our read‐based computational microbiome function annotation tool. Our results reveal a more diverse microbiome functional capacity and activity in the early‐ vs. late‐spring samples. We also find that functional dissimilarity between the same‐sample metagenomes and metatranscriptomes is significantly higher in early than late spring samples. These findings suggest that early spring samples may contain a larger fraction of DNA of dormant (or dead) organisms, while late spring samples reflect a new, metabolically active community. We further show that the abundance of sequencing reads mapping to the fatty acid synthesis‐related microbial pathways in late spring metagenomes and metatranscriptomes is significantly correlated with the organic acid levels measured in these samples. Similarly, the organic acid levels correlate with the pathway read abundances of geraniol degradation and inversely correlate with those of styrene degradation, suggesting a possible nutrient change. Our study thus highlights the activity of microbial degradation pathways of complex organic compounds previously unreported at low temperatures.

## Graphical abstract

Computational analysis of metagenomic and metatranscriptomic data from snow samples collected at different springtimes in Svalbard, Norway suggests that a new microbial community is metabolically active in the late spring, while early spring samples likely overwhelmingly carry genomic residue from prior seasons. Furthermore, the correlation between the sequencing‐based, computationally determined metabolic pathways, and the experimentally measured organic acid concentration highlights active microbial degradation of complex organic compounds previously unreported at low temperatures.

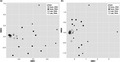

## 1 INTRODUCTION

Abiotic parameters, such as temperature, pH, and pressure, create stress on microorganisms, especially in extreme environments (Rothschild & Mancinelli, 2001). The cryosphere, an extreme cold environment, covers a large portion of Earth's surface. Over 14% of the world's biosphere is located at the planetary poles, while 90% by volume of the ocean is colder than 5°C (Price & Sowers, 2004). Taxonomic surveys based on 16S rRNA gene sequencing have described significant microbial diversity in glacial ice (Cameron et al., 2016; Christner, Mosley‐Thompson, Thompson, & Reeve, 2001; Christner et al., 2000), cryoconite (Uetake et al., 2016; Webster‐Brown, Hawes, Jungblut, Wood, & Christenson, 2015), sea ice (Brinkmeyer et al., 2003), and polar and alpine snow (Amato et al., 2007; Harding, Jungblut, Lovejoy, & Vincent, 2011; Larose et al., 2010; Maccario, Carpenter, Deming, Vogel, & Larose, 2019; Wunderlin, Ferrari, & Power, 2016). Bacteria seem to be ubiquitous in the snow and belong to numerous taxa such as *Proteobacteria* (*Alpha*‐, *Beta*‐, and *Gamma*‐), the *Cytophaga–Flexibacter–Bacteroides* group, *Actinobacteria*, and *Cyanobacteria* (Harding et al., 2011; Larose et al., 2010, 2013; Segawa et al., 2005), although their reported populations vary based on season, sampling location, and analysis methods. For example, the diversity of organisms in the snow from the Canadian high Arctic ice sheet was 20 times lower than that measured in Tibetan plateau snow (Harding et al., 2011; Zhang, Yang, Wang, & Hou, 2010), which may reflect the real community or methodological differences. A variety of approaches, such as cultivation, ribosomal profiling, and stable isotope probing, have been used to detect and measure microbial activity at subzero temperatures in permafrost soils; for review, see Nikrad, Kerkhof, and Haggblom (2016). While these offer insights into the microbial interactions within the *soil* environment in the cold, considerably less is known about the specifics of microorganism functionality in the *snow*. One pioneering metagenomic study correlated microbiome functionality with chemical parameters, such as mercury concentration in the Arctic spring snow samples (Maccario, Vogel, & Larose, 2014). Another notes that biological activity in the snow is a poorly constrained source and potential modifier of organic compounds (Ariya et al., 2011). Thus, organisms active in the snow may be involved in a range of processes involving organic matter, potentially impacting atmospheric and biogeochemical cycles (McNeil, 2012).

Large scale metagenome sequencing drastically increased the publicly available metagenomic data from high profile projects such as Terragenome (Vogel et al., 2009), the Global Ocean Sampling Expedition (Rusch et al., 2007), the Human Microbiome Project (Human Microbiome Project Consortium, 2012), and Tara Oceans (Sunagawa et al., 2015). Studies have been carried out in a wide variety of environments including the human gut (Gevers et al., 2014; Qin et al., 2012; Zhu et al., 2018), groundwater (Hemme et al., 2015), acid mine drainage (Chen et al., 2014), beach sand (Rodriguez‐R et al., 2015), etc., and identified potential diagnostic, therapeutic, or bioremediation targets. With ample data, comparative analysis of metagenomes/metatranscriptomes under different conditions highlights the key microbial members and their molecular functions that result from and/or contribute to niche differences (Zhu, Delmont, Vogel, & Bromberg, 2015; Zhu, Mahlich, Miller, & Bromberg, 2018). While such analyses have not yet been widely applied to cold environment samples, they could help elucidate microbial mechanisms of survival and adaptation at low temperatures.

Bergk‐Pinto et al. studied the microbial ecology in 20 snow samples collected during early and late spring (mid‐April to mid‐June, 2011) in Svalbard, Norway (Bergk Pinto, Maccario, Dommergue, Vogel, & Larose, 2019). Using a combined method of marker genes and network analysis, the study revealed that the snow microbial community shifted from early spring cooperation to late spring competition, accompanied by enrichment in antibiotic resistance genes (Bergk Pinto et al., 2019). Here, we further analyzed these samples to investigate the microbial metabolism of organic compounds at low temperatures. We annotated the sample metagenomic and metatranscriptomic data using mi‐faser (microbiome functional annotation of sequencing reads) (Zhu et al., 2018). This bioinformatic tool provides high accuracy (>90%) functional annotation of sequencing reads, using a reference database of experimentally verified microbial enzymes. Our results highlighted significantly lower metagenome‐to‐metatranscriptome similarity in the early spring than in the late spring samples. We also found that in the late spring samples, the abundance of sequencing reads mapping to the components of the fatty acid synthesis‐related microbial pathways significantly correlated with the experimentally determined levels of organic acids. We further observed that the rise in organic acid levels correlated with the enrichment of the geraniol degradation pathway use and the depletion of the styrene degradation pathway. This finding might represent a change in nutrient conditions during the community growth period. To summarize, here we observed microbial functionality necessary for the degradation of complex organic compounds in both metagenomes and metatranscriptomes of the late spring snow samples. Our results thus offer new evidence for presence of these microbial activities at temperatures below 0°C.

## 2 MATERIALS AND METHODS

### 2.1 Data collection and preprocessing

We obtained the metagenomic, metatranscriptomic, and chemistry (organic acid) data for 20 snow samples from the Environmental Microbial Genomics Group (see Data Availability Statement). The technical details of sampling and sequencing are described in Bergk Pinto et al. (2019). Briefly, snow was collected over two months from mid‐April to mid‐June at Ny Ålesund on the Spitsbergen island of Svalbard, Norway (78°56′N, 11°52′E). The field site, a 50 m^2^ perimeter with restricted access (to reduce contamination from human sources), is located along the south coast of the Kongsfjorden, which is oriented SE‐NW and open to the sea on the west side. The site is in a bird sanctuary (for migratory birds that generally arrive in June) and the closest building is located 1 km away. Surface snow layers (2−3 L) from the field site were collected into sterile Whirl‐Pak bags using a sterilized Teflon shovel. The samples for chemistry analysis were stored frozen and shipped back to France. Microbiology samples were processed immediately after collection in a field laboratory. Specifically, samples were left to melt at room temperature (~8 hr) and filtered onto sterile 0.22 µM 47 mm filters (Millipore) using a sterile filtration unit (Nalge Nunc International Corporation) as soon as they were completely melted. Filters were stored in Eppendorf tubes at −20°C for sequencing and further analysis. We note that melting at room temperature may have biased our microbiome expression (metatranscriptome) observations. However, we also note that the bias introduced by warming sample temperatures would have equally impacted late and early spring samples, suggesting that their differences are still a reliable source of functional evidence. Details on sampling conditions, sample site, and chemical analyses can also be found in Bergk Pinto et al. (2019). Sequencing data were quality filtered using Mothur (Schloss et al., 2009) with settings described in Schloss, Gevers, and Westcott (2011). Base overrepresentation was controlled using FastQC (Andrews, 2010). Usearch (Edgar, 2010) was used to identify and remove remaining adaptors.

### 2.2 Analysis

The post‐quality‐control reads were submitted to mi‐faser web service (Miller, Zhu, & Bromberg, 2017; Zhu et al., 2018) for annotation. For each sample, mi‐faser returns a read abundance table of enzyme functionality detected in the sample, that is, the EC profile (EC stands for Enzyme Commission (1992)). For all further analysis, read abundance was standardized by the total number of reads in each sample. To create the pathway profile of a sample, for each known KEGG functional pathway (Kanehisa, Sato, Kawashima, Furumichi, & Tanabe, 2016), we divided the sum of the reads mapping to all enzymes in this pathway by the total number of enzymes in this pathway. The NMDS diagrams were generated with the (enzyme and pathway) profiles of samples assigned to four groups, early_DNA (early spring metagenomes), early_RNA (early spring metatranscriptomes), late_DNA (late spring metagenomes), and late_RNA (late spring metatranscriptomes). The Euclidean distances between the same‐sample DNA and RNA NMDS points were calculated and compared across the four groups. The significance of differences in distance distributions was evaluated using a two‐tailed *t* test at 0.05 threshold. Organic acid levels were standardized across all samples to the sum total of their abundances in all samples. The Spearman correlation coefficients, as well as the significance of correlations, were calculated by the R function cor.test with algorithm AS89 (Best & Roberts, 1975).

## 3 RESULTS AND DISCUSSION

### 3.1 Early to late spring dissimilarity and metagenome‐to‐transcriptome divergence highlight community activity in late spring samples

**FIGURE 1:**
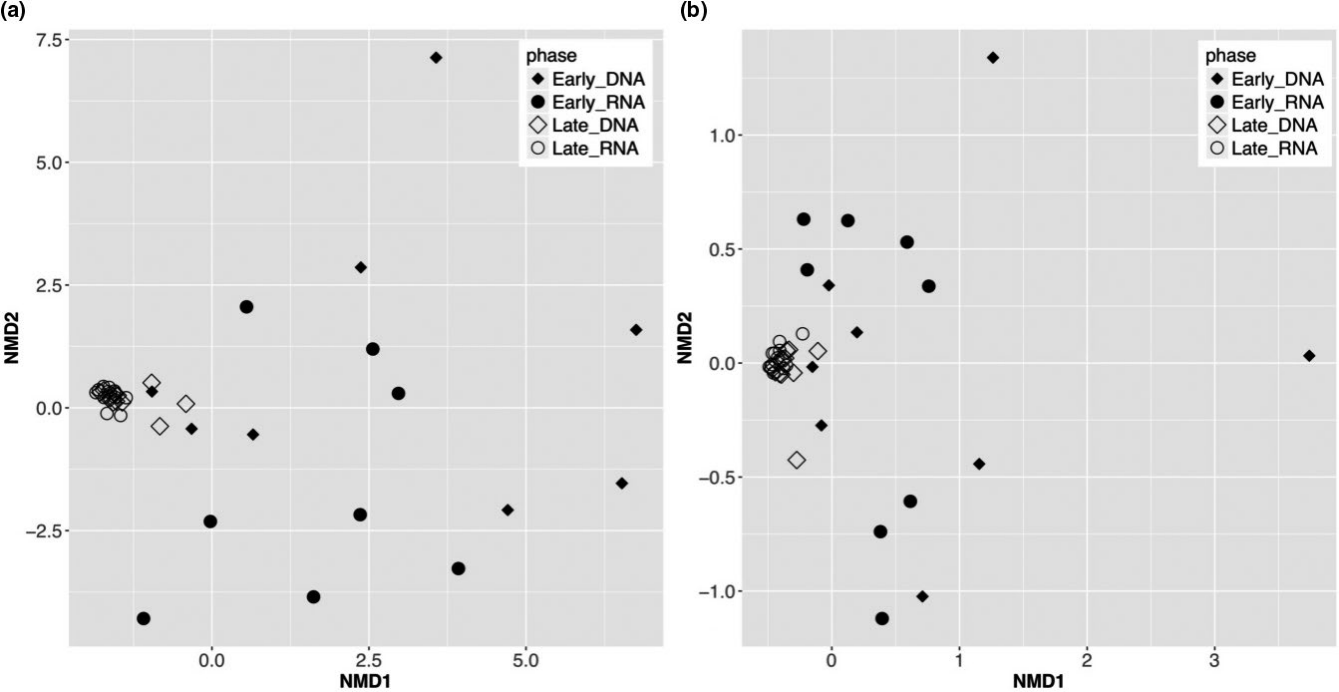
NMDS suggests higher microbial functional beta‐diversity in early spring samples than in late spring ones. The average Euclidean inter‐sample distance between (a) sample EC profiles is 4.8 ± 2.3 for early spring samples, and 0.4 ± 0.3 for late spring samples and (b) sample pathway profiles is 1.4 ± 0.9 for early spring samples and 0.1 ± 0.1 for late spring samples. Intuitively, observe that early spring samples are widely distributed in both panels, while late spring samples tend to concentrate

**FIGURE 2:**
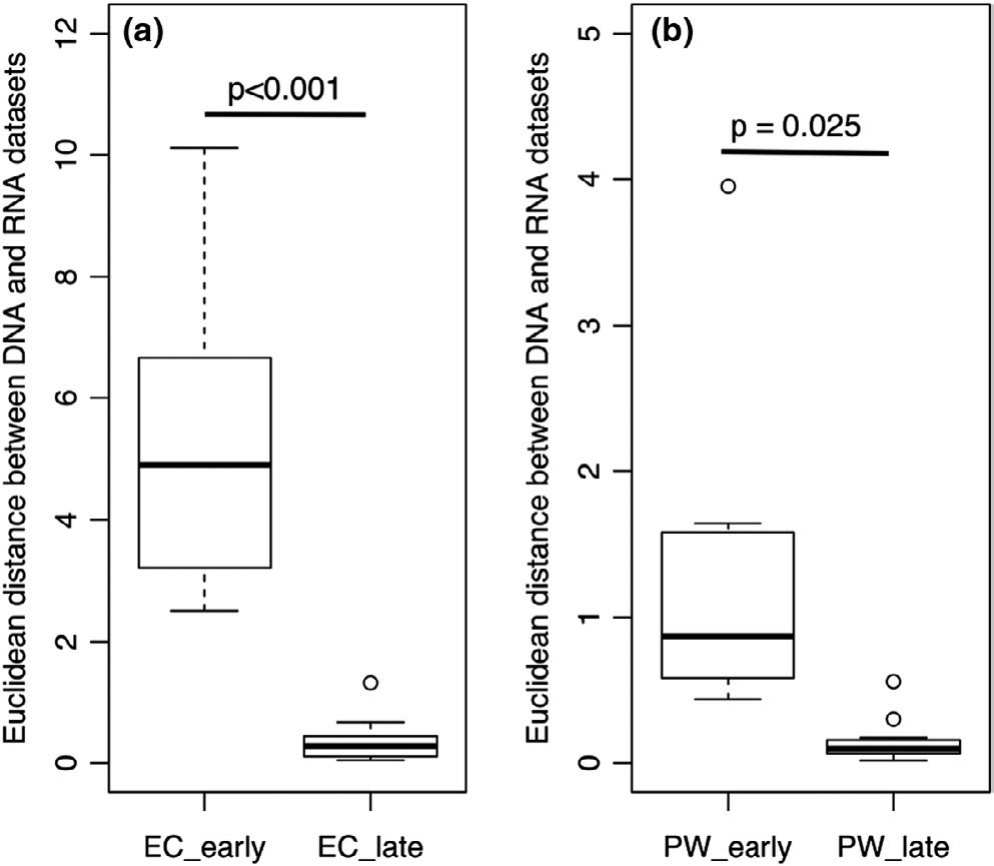
Metagenomes and metatranscriptomes of the same sample are significantly more similar in late than early spring samples. The distribution boxplots of the distance between the DNA and RNA sample (a) EC and (b) pathway profiles. Note that difference is less significant for pathway profiles

**FIGURE A1:**
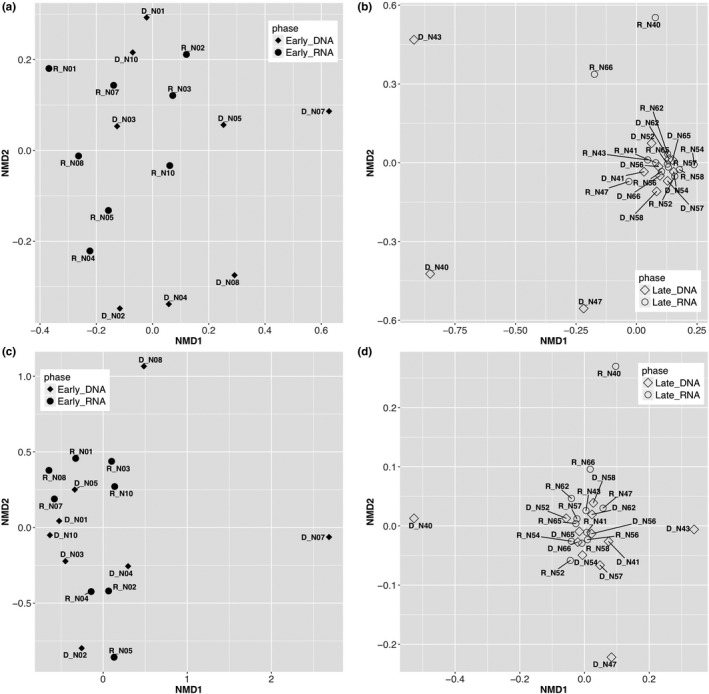
NMDS plots of (a) EC profiles of early spring samples, (b) EC profiles of late spring samples, (c) pathway profiles of early spring samples, and (d) pathway profiles of late spring samples

While the metagenome reflects the potential function of a microbial community, metatranscriptomic analyses reflect genes that are transcribed, highlighting the implicitly active fraction of these functions. In analyzing the metagenomes and metatranscriptomes of early and late spring polar snow samples, we observed that (a) the early spring samples were more diverse than the late spring samples in both potential and active microbial functionality (measured as the Euclidean distance between entries on the NMDS plot; Materials and Methods; EC profile sample distance: early spring = 4.8 ± 2.3, late spring = 0.4 ± 0.3, Figure 1a, Figure A1a,b; pathway profile sample distance: early spring = 1.4 ± 0.9, late spring = 0.1 ± 0.1, Figure 1b, Figure A1c,d) and that (b) metagenome‐to‐metatranscriptome similarity of the same sample was significantly lower in early than in late spring (in both comparisons of the EC profiles, *t* test *p*‐value <0.001, Figure 2a and the pathway profiles, *t* test *p*‐value = 0.025, Figure 2b). Note that for all comparisons, ~29% ECs (195 of 683) in our data could not be mapped to known KEGG pathways (Appendix B at https://doi.org/10.6084/m9.figshare.12290711).

The discrepancy in functional annotation of metagenomes (DNA) and metatranscriptomes (cDNA) of the same samples has previously been observed in environments such as the human gut (Franzosa et al., 2014) and open ocean (Shi, Tyson, Eppley, & DeLong, 2010). The genes observed in the metagenomes represent potential functions that may or may not be expressed in the environment at the time of sampling and could belong to inactive community members. The metatranscriptome‐specific functions, on the other hand, belong to active members of the community at the time of sampling (Yu & Zhang, 2012). The exceedingly low metagenome‐to‐metatranscriptome similarity (high distance/dissimilarity) in the early spring samples (Figure 2) suggests that the active members (organisms and molecular functions) in early spring occur at such low abundance that metagenomic sequencing fails to detect them. We speculated that the potential functional diversity in the early spring metagenome samples (Figure 1; Figure A1; DNA datasets) might come from the DNA of dead or inactive cells preserved in the snow. Interestingly, many microorganisms identified in snow and ice via 16S rRNA gene surveys are non‐psychrophiles (Cowan & Tow, 2004) and their membership in the community needs further investigation. Meanwhile, the diversity of active microbial functionality in the early spring metatranscriptomes (Figure 1; Figure A1; RNA datasets) reflected diverse microbial activities (238 enzymatic functions involved in 84 metabolic pathways including cell size reduction, changes in fatty acid and phospholipid membrane composition, and decrease in the fractional volume of cellular water). This observation is in line with the known variety of survival strategies employed by microbes at low temperature (Nikrad et al., 2016; Price & Sowers, 2004).

With the warming in the late spring, the active community made up a larger fraction of the sequenced reads and, thus, manifested in more homogeneity. Previous 16S rRNA‐based taxonomic analysis on the same dataset also observed a shift in the community from early to late spring (Bergk Pinto et al., 2019). While the early spring samples contained a core community of 59 OTUs, there were only 29 OTUs in the late spring samples, with 42 early spring core OTUs disappearing from the core community of late spring samples (and 12 late spring‐specific OTUs appearing) (Bergk Pinto et al., 2019). The early spring community thus contained a higher diversity of organisms of which only a small fraction was likely active; the inactive community members could no longer be detected in the late spring samples. As a result, we observed a decrease in functional diversity (Figure 1; Figure A1) and an increase in the metagenome‐to‐metatranscriptome similarity (Figure 2). Also, our result suggests that despite the taxonomic diversity in the late spring samples, their functional potential and activity were highly similar (Figure 1; Figure A1), highlighting the advantages of functional analyses to the 16S rRNA gene surveys.

### 3.2 Microbial use of complex organic compounds in the snow

**FIGURE A2:**
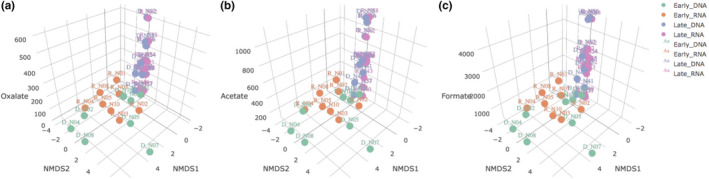
Increased levels of (a) oxalate, (b) acetate, and (c) formate in the late spring samples. *X*‐ and *Y*‐axis represent the NMDS dimensions; *Z*‐axis represents the organic acid level in nmol/kg

Snow provides a medium and nutrients for microbial growth and associated physicochemical processes (Domine & Shepson, 2002); growth implies the utilization of nutrients. In glacial ice metagenomes, numerous genes related to xenobiotics, biopolymers, and other carbon sources were detected, suggesting that ice microorganisms have the potential to degrade a wide range of substrates (Stibal, Šabacká, & Žárský, 2012). The levels of all three organic acids (oxalate, acetate, and formate) measured in our study remained in low concentration in the early spring samples (Appendix C: https://doi.org/10.6084/m9.figshare.12290720). They increased in the late spring (Figure A2), possibly concomitant with increased microbial activity. Increased activity of microbial community members in the late spring snow might thus be related to the changes in organic acid levels in the samples.

**FIGURE A3:**
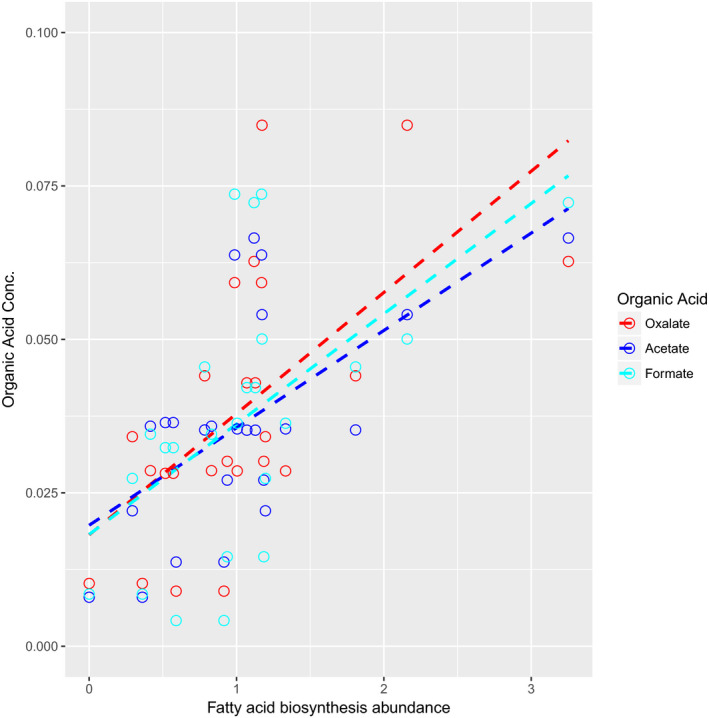
Fatty acid biosynthesis functional abundance correlates with organic acid levels. The pathway abundance is in standardized read‐count units, that is, the sum of the reads mapped by mi‐faser to all enzymes in this pathway standardized and rescaled by (1) the pathway size, that is, the total number of enzymes in this pathway and (2) the sample size, that is, the total number of reads in the sequencing sample. The dashed lines are simply visual aids of the data correlation

**FIGURE A4:**
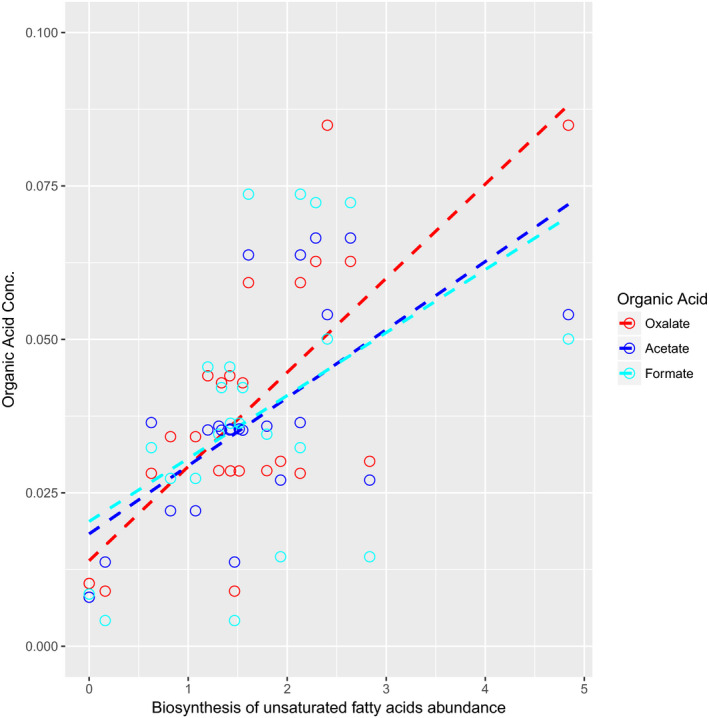
Biosynthesis of unsaturated fatty acids functional abundance correlates with organic acid levels. The pathway abundance is in standardized read‐count units, that is, the sum of the reads mapped by mi‐faser to all enzymes in this pathway standardized and rescaled by (1) the pathway size, that is, the total number of enzymes in this pathway and (2) the sample size, that is, the total number of reads in the sequencing sample. The dashed lines are simply visual aids of the data correlation

**FIGURE A5:**
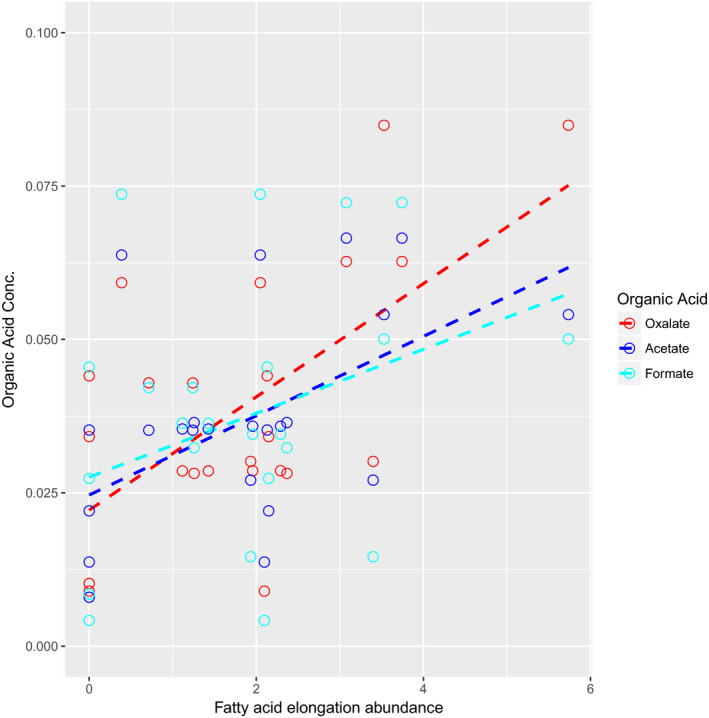
Fatty acid elongation functional abundance correlates with organic acid levels. The pathway abundance is in standardized read‐count units, that is, the sum of the reads mapped by mi‐faser to all enzymes in this pathway standardized and rescaled by (1) the pathway size, that is, the total number of enzymes in this pathway and (2) the sample size, that is, the total number of reads in the sequencing sample. The dashed lines are simply visual aids of the data correlation

**FIGURE A6:**
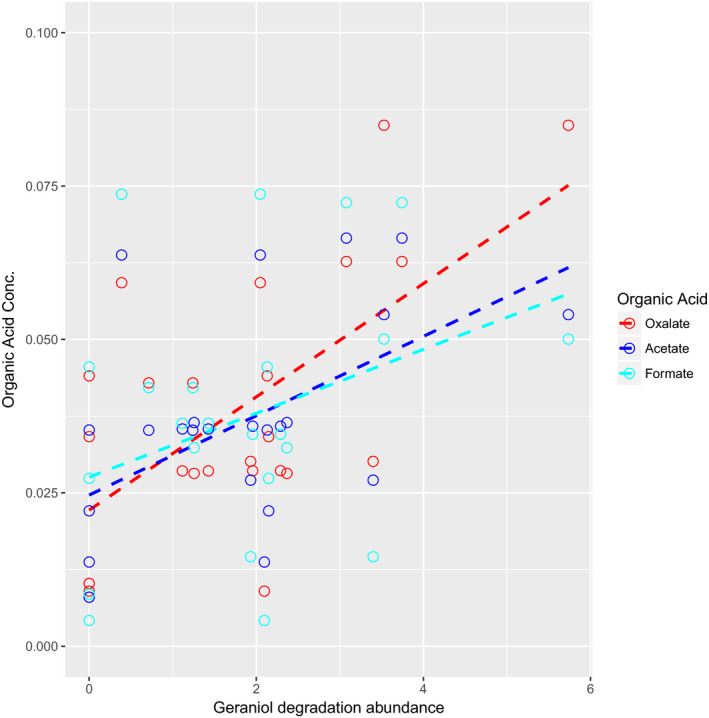
Geraniol degradation functional abundance correlates with organic acid levels. The pathway abundance is in standardized read‐count units, that is, the sum of the reads mapped by mi‐faser to all enzymes in this pathway standardized and rescaled by (1) the pathway size, that is, the total number of enzymes in this pathway and (2) the sample size, that is, the total number of reads in the sequencing sample. The dashed lines are simply visual aids of the data correlation

**FIGURE A7:**
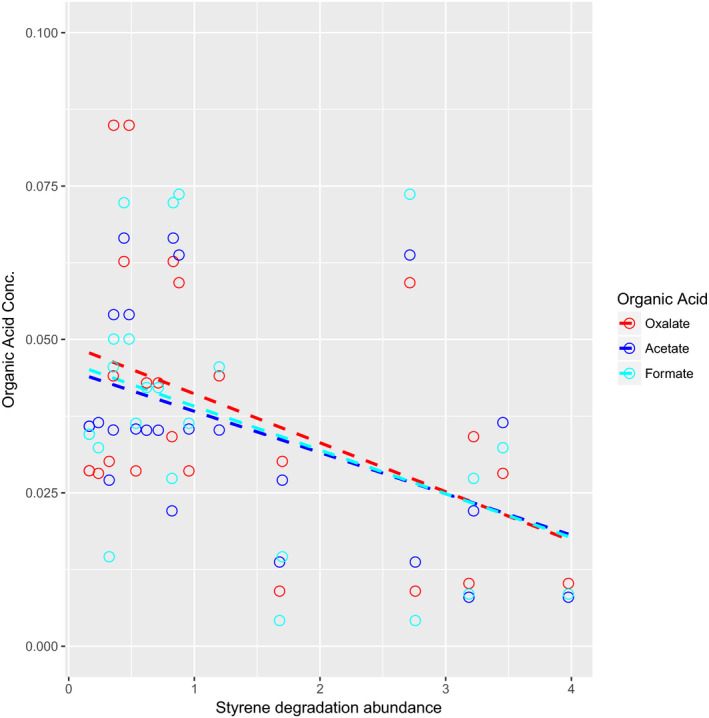
Styrene degradation functional abundance correlates with organic acid levels. The pathway abundance is in standardized read‐count units, that is, the sum of the reads mapped by mi‐faser to all enzymes in this pathway standardized and rescaled by (1) the pathway size, that is, the total number of enzymes in this pathway and (2) the sample size, that is, the total number of reads in the sequencing sample. The dashed lines are simply visual aids of the data correlation

Microbial preferences for different carbon classes were studied in Antarctic snow, showing a higher rate of carbon uptake when snow microcosms were amended with a combination of simple and complex carbon sources (Antony et al., 2012). The appearance of organic acids in the snow may have both abiotic (e.g., aerial deposition) and biotic (e.g., microbial activity) origins. In our study, the clear correlation (co‐interia (DolÉDec & Chessel, 1994)) of organic acid concentrations with microbial activity levels, captured by metatranscriptomes, strongly indicated active metabolism in the late spring samples (Table 1; Figures A3, A4, A5, A6, A7). Note that both mi‐faser and EggNog Mapper (Huerta‐Cepas et al., 2017) functional profiles recognized this correlation (Table A1), albeit mi‐faser reached a higher level of significance.

**TABLE 1:** The metagenomic/metatranscriptomic pathways significantly correlate (Spearman's *ρ*; *p*‐value <0.05) with the levels of organic acids in the late spring samples

Pathway	Oxalate		Acetate		Formate	
	*ρ*	*p*‐value	*ρ*	*p*‐value	*ρ*	*p*‐value
Fatty acid biosynthesis	**0.63**	**0.001**	**0.42**	**0.040**	**0.56**	**0.004**
Biosynthesis of unsaturated fatty acids	**0.61**	**0.002**	**0.66**	**0.004**	**0.55**	**0.005**
Fatty acid elongation	**0.46**	**0.025**	**0.55**	**0.005**	0.34	0.102
Geraniol degradation	**0.46**	**0.025**	**0.55**	**0.005**	0.34	0.102
Styrene degradation	−0.33	0.116	**−0.42**	**0.041**	−0.33	0.110

Bold values indicate *p* < .05.

Among the enzymes that were not mapped to known KEGG pathways, two tRNA‐methyltransferases (2.1.1.61 and 2.1.1.217; *p*‐value <0.05, Materials and Methods) showed a significant correlation with organic acid levels. tRNA methylation regulates important steps in protein synthesis and is essential for microbial growth in high temperature (Hori, 2014). Our results suggest that it could be also important in low‐temperature conditions.

To summarize, we identified five pathways in our metagenomes/metatranscriptomes that significantly correlated with organic acid levels in the late spring samples (*p*‐value <0.05 highlighted in bold in Table 1; Figures A3, A4, A5, A6, A7; Materials and Methods): fatty acid biosynthesis, biosynthesis of unsaturated fatty acids, fatty acid elongation, geraniol degradation, and styrene degradation. The top three pathways were related to fatty acid synthesis and elongation. Fatty acids are essential due to their role in membrane synthesis and critical in low temperatures that affect membrane fluidity (Cronan & Thomas, 2009). The following degradation pathways were also important. Geraniol is a terpene produced by a variety of plants for its antibacterial activities (Friedman, Henika, & Mandrell, 2002). Terpenes are released from plants (Marmulla & Harder, 2014) and deposited in arctic snowpacks like other volatile organic compounds (Kos, Kanthasami, Adechina, & Ariya, 2014). Geraniol degradation allows some bacteria, for example *Pseudomonas putida*, to utilize geraniol as their sole carbon and energy source (Vandenbergh & Wright, 1983). *Pseudomonas putida* is also known to degrade styrene (O'Connor, Duetz, Wind, & Dobson, 1996) and polystyrene (Ward, Goff, Donner, Kaminsky, & O'Connor, 2006). Therefore, the organic acid level correlation (with geraniol degradation) and anticorrelation (with styrene degradation) may suggest a change of nutrient availability in the environment. *Pseudomonas putida* is known to possess diverse metabolic capabilities to degrade a variety of organic solvents. Most of its strains are mesophilic, but one (KT2440) has been reported as psychrotolerant (optimal growth at 30°C but can proliferate at 4°C) (Fonseca, Moreno, & Rojo, 2011). To the best of our knowledge, no microbial metabolism of geraniol and styrene has been reported at low temperatures. Our functional omics study thus provides new evidence suggestive of active microbial degradation of complex organic compounds at subzero temperatures.

## 4 CONCLUSIONS

We defined microbial activity at low temperatures as the gene abundance level in metagenomic and metatranscriptomic datasets from snow in early and late spring. Our results highlight the novel microbial activity of complex organic compound degradation at low temperatures. A further in‐depth exploration of the functionality of the cryosphere inhabitants can contribute to our understanding of microbial metabolism at low temperatures and aid in the discovery of novel enzymes with potential industrial and bioremediation value.

## CONFLICTS OF INTEREST

None declared.

## AUTHOR CONTRIBUTIONS

**Chengsheng Zhu:** Conceptualization (lead); formal analysis (lead); methodology (lead); visualization (lead); writing – original draft (lead); writing – review & editing (lead). **Maximilian Miller:** Software (lead). **Nicholas Lusskin:** Software (supporting). **Benoît Bergk Pinto:** Data curation (equal); writing – review & editing (supporting). **Lorrie Maccario:** Data curation (equal); writing – review & editing (supporting). **Max Häggblom:** Writing – review & editing (supporting). **Timothy Vogel:** Writing – review & editing (supporting). **Catherine Larose:** Writing – review & editing (supporting). **Yana Bromberg:** Conceptualization (equal); resources (lead); supervision (lead); writing – review & editing (lead).

## ETHICS STATEMENT

None required.

## Data Availability

The associated data and materials are accessible via figshare: metagenomic and metatranscriptomic data: https://doi.org/10.6084/m9.figshare.12330560; correlation code: https://doi.org/10.6084/m9.figshare.12290771 ; pathway profile: https://doi.org/10.6084/m9.figshare.12290750; Appendix B (ECs that were not mapped to KEGG pathways): https://doi.org/10.6084/m9.figshare.12290711; Appendix C (Organic acid levels in early and late spring samples): https://doi.org/10.6084/m9.figshare.12290720).

## APPENDIX A

**TABLE A1:** Co‐inertia results comparing the Eggnog mapper vs. mi‐faser

Mapper	Annotation	Metagenomes		Metatranscriptomes	
		Co‐inertia RV	*p*‐value	Co‐inertia RV	*p*‐value
Eggnog mapper	Genes id	0.44	0.033^*^	0.43	0.072
	GO terms	0.45	0.003^**^	0.44	0.023^*^
	KEGG pathways	0.59	0.0002^***^	0.37	0.064
mi‐faser	EC	0.51	0.0009^***^	0.5	0.001^**^
	KEGG pathways	0.48	0.0003^***^	0.49	0.0004^***^

Note

Co‐inertia of organic acid levels with the Eggnog mapper or mi‐faser abundances. The co‐inertia analysis measures the co‐variance of the organic acid levels and the functional profile abundances determined by Eggnog mapper and mi‐faser. The significance of each co‐inertia was tested using a permutation test (10,000 permutations).

* *p* < .05.

** *p* < .01.

*** *p* < .001.

## References

[mbo31100-bib-0001] Amato, P. , Hennebelle, R. Ã. , Magand, O. , Sancelme, M. , Delort, A.‐M. , Barbante, C. , … Ferrari, C. (2007). Bacterial characterization of the snow cover at Spitzberg, Svalbard. FEMS Microbiology Ecology, 59(2), 255–264. 17328766 10.1111/j.1574-6941.2006.00198.x

[mbo31100-bib-0002] Andrews, S. (2010). FastQC: A quality control tool for high throughput sequence data.

[mbo31100-bib-0003] Antony, R. , Krishnan, K. P. , Laluraj, C. M. , Thamban, M. , Dhakephalkar, P. K. , Engineer, A. S. , & Shivaji, S. (2012). Diversity and physiology of culturable bacteria associated with a coastal Antarctic ice core. Microbiological Research, 167(6), 372–380. 22537873 10.1016/j.micres.2012.03.003

[mbo31100-bib-0004] Ariya, P. A. , Domine, F. , Kos, G. , Amyot, M. , Côté, V. , Vali, H. , … Mortazavi, R. (2011). Snow – A photobiochemical exchange platform for volatile and semi‐volatile organic compounds with the atmosphere. Environmental Chemistry, 8(1), 62–73.

[mbo31100-bib-0005] Bergk Pinto, B. , Maccario, L. , Dommergue, A. , Vogel, T. M. , & Larose, C. (2019). Do organic substrates drive microbial community interactions in Arctic snow? Frontiers in Microbiology, 10 10.3389/fmicb.2019.02492. [Epub ahead of print]. PMC6842950 31749784

[mbo31100-bib-0006] Best, D. J. , & Roberts, D. E. (1975). Algorithm AS 89: The upper tail probabilities of Spearman's Rho. Applied Statistics, 24, 377–379.

[mbo31100-bib-0007] Brinkmeyer, R. , Knittel, K. , Jürgens, J. , Weyland, H. , Amann, R. , & Helmke, E. (2003). Diversity and structure of bacterial communities in Arctic versus Antarctic pack ice. Applied and Environmental Microbiology, 69(11), 6610–6619. 14602620 10.1128/AEM.69.11.6610-6619.2003 PMC262250

[mbo31100-bib-0008] Cameron, K. A. , Stibal, M. , Zarsky, J. D. , Gözdereliler, E. , Schostag, M. , & Jacobsen, C. S. (2016). Supraglacial bacterial community structures vary across the Greenland ice sheet. FEMS Microbiology Ecology, 92(2), fiv164. 26691594 10.1093/femsec/fiv164

[mbo31100-bib-0009] Chen, L.‐X.‐,. Hu, M. , Huang, L. , Hua, Z. , Kuang, J. , Li, S. , & Shu, W. (2014). Comparative metagenomic and metatranscriptomic analyses of microbial communities in acid mine drainage. The ISME Journal, 9, 1579. 25535937 10.1038/ismej.2014.245 PMC4478699

[mbo31100-bib-0010] Christner, B. C. , Mosley‐Thompson, E. , Thompson, L. G. , & Reeve, J. N. (2001). Isolation of bacteria and 16S rDNAs from Lake Vostok accretion ice. Environmental Microbiology, 3(9), 570–577. 11683867 10.1046/j.1462-2920.2001.00226.x

[mbo31100-bib-0011] Christner, B. C. , Mosley‐Thompson, E. , Thompson, L. G. , Zagorodnov, V. , Sandman, K. , & Reeve, J. N. (2000). Recovery and identification of viable bacteria immured in glacial ice. Icarus, 144(2), 479–485.

[mbo31100-bib-0012] Cowan, D. A. , & Tow, L. A. (2004). Endangered Antarctic environments. Annual Review of Microbiology, 58(1), 649–690. 10.1146/annurev.micro.57.030502.090811 15487951

[mbo31100-bib-0013] Cronan, J. E. , & Thomas, J. (2009). Bacterial fatty acid synthesis and its relationships with polyketide synthetic pathways. Methods in Enzymology, 459, 395–433. 19362649 10.1016/S0076-6879(09)04617-5 PMC4095770

[mbo31100-bib-0014] DolÉDec, S. , & Chessel, D. (1994). Co‐inertia analysis: An alternative method for studying species–environment relationships. Freshwater Biology, 31(3), 277–294.

[mbo31100-bib-0015] Domine, F. , & Shepson, P. B. (2002). Air‐snow interactions and atmospheric chemistry. Science, 297(5586), 1506–1510. 12202818 10.1126/science.1074610

[mbo31100-bib-0016] EC (1992). Enzyme nomenclature 1992: Recommendations of the Nomenclature Committee of the International Union of Biochemistry and Molecular Biology on the nomenclature and classification of enzymes. San Diego, CA: Academic Press.

[mbo31100-bib-0017] Edgar, R. C. (2010). Search and clustering orders of magnitude faster than BLAST. Bioinformatics, 26(19), 2460–2461. 20709691 10.1093/bioinformatics/btq461

[mbo31100-bib-0018] Fonseca, P. , Moreno, R. , & Rojo, F. (2011). Growth of *Pseudomonas putida* at low temperature: Global transcriptomic and proteomic analyses. Environmental Microbiology Reports, 3(3), 329–339. 23761279 10.1111/j.1758-2229.2010.00229.x

[mbo31100-bib-0019] Franzosa, E. A. , Morgan, X. C. , Segata, N. , Waldron, L. , Reyes, J. , Earl, A. M. , … Huttenhower, C. (2014). Relating the metatranscriptome and metagenome of the human gut. Proceedings of the National Academy of Sciences, 111(22), E2329–E2338. 10.1073/pnas.1319284111 PMC4050606 24843156

[mbo31100-bib-0020] Friedman, M. , Henika, P. R. , & Mandrell, R. E. (2002). Bactericidal activities of plant essential oils and some of their isolated constituents against *Campylobacter jejuni*, *Escherichia coli*, *Listeria monocytogenes*, and *Salmonella enterica* . Journal of Food Protection, 65(10), 1545–1560. 12380738 10.4315/0362-028x-65.10.1545

[mbo31100-bib-0021] Gevers, D. , Kugathasan, S. , Denson, L. A. , Vázquez‐Baeza, Y. , Van Treuren, W. , Ren, B. , … Xavier, R. J. (2014). The treatment‐naive microbiome in new‐onset Crohn's disease. Cell Host & Microbe, 15(3), 382–392. 24629344 10.1016/j.chom.2014.02.005 PMC4059512

[mbo31100-bib-0022] Harding, T. , Jungblut, A. D. , Lovejoy, C. , & Vincent, W. F. (2011). Microbes in high Arctic snow and implications for the cold biosphere. Applied and Environmental Microbiology, 77(10), 3234. 21460114 10.1128/AEM.02611-10 PMC3126466

[mbo31100-bib-0023] Hemme, C. L. , Tu, Q. , Shi, Z. , Qin, Y. , Gao, W. , Deng, Y. E. , … Zhou, J. (2015). Comparative metagenomics reveals impact of contaminants on groundwater microbiomes. Frontiers in Microbiology, 6 10.3389/fmicb.2015.01205. [Epub ahead of print]. PMC4628106 26583008

[mbo31100-bib-0024] Hori, H. (2014). Methylated nucleosides in tRNA and tRNA methyltransferases. Frontiers in Genetics, 5, 144. 24904644 10.3389/fgene.2014.00144 PMC4033218

[mbo31100-bib-0025] Huerta‐Cepas, J. , Forslund, K. , Coelho, L. P. , Szklarczyk, D. , Jensen, L. J. , von Mering, C. , & Bork, P. (2017). Fast genome‐wide functional annotation through orthology assignment by eggNOG‐Mapper. Molecular Biology Evolution, 34(8), 2115–2122. 28460117 10.1093/molbev/msx148 PMC5850834

[mbo31100-bib-0026] Human Microbiome Project Consortium (2012). Structure, function and diversity of the healthy human microbiome. Nature, 486(7402), 207–214. 22699609 10.1038/nature11234 PMC3564958

[mbo31100-bib-0027] Kanehisa, M. , Sato, Y. , Kawashima, M. , Furumichi, M. , & Tanabe, M. (2016). KEGG as a reference resource for gene and protein annotation. Nucleic Acids Research, 44(D1), D457–D462. 10.1093/nar/gkv1070 26476454 PMC4702792

[mbo31100-bib-0028] Kos, G. , Kanthasami, V. , Adechina, N. , & Ariya, P. A. (2014). Volatile organic compounds in Arctic snow: Concentrations and implications for atmospheric processes. Environmental Science: Processes & Impacts, 16(11), 2592–2603. 25249335 10.1039/c4em00410h

[mbo31100-bib-0029] Larose, C. , Berger, S. , Ferrari, C. , Navarro, E. , Dommergue, A. , Schneider, D. , & Vogel, T. M. (2010). Microbial sequences retrieved from environmental samples from seasonal arctic snow and meltwater from Svalbard, Norway. Extremophiles, 14(2), 205–212. 20066448 10.1007/s00792-009-0299-2

[mbo31100-bib-0030] Larose, C. , Prestat, E. , Cecillon, S. , Berger, S. , Malandain, C. , Lyon, D. , … Vogel, T. M. (2013). Interactions between snow chemistry, mercury inputs and microbial population dynamics in an Arctic snowpack. PLoS One, 8(11), e79972. 24282515 10.1371/journal.pone.0079972 PMC3839931

[mbo31100-bib-0031] Maccario, L. , Carpenter, S. D. , Deming, J. W. , Vogel, T. M. , & Larose, C. (2019). Sources and selection of snow‐specific microbial communities in a Greenlandic sea ice snow cover. Scientific Reports, 9(1), 2290. 30783153 10.1038/s41598-019-38744-y PMC6381142

[mbo31100-bib-0032] Maccario, L. , Vogel, T. M. , & Larose, C. (2014). Potential drivers of microbial community structure and function in Arctic spring snow. Frontiers in Microbiology, 5, 413. 25147550 10.3389/fmicb.2014.00413 PMC4124603

[mbo31100-bib-0033] Marmulla, R. , & Harder, J. (2014). Microbial monoterpene transformations‐a review. Frontiers in Microbiology, 5, 346. 25076942 10.3389/fmicb.2014.00346 PMC4097962

[mbo31100-bib-0034] McNeil, A. J. (2012). PROFILE: Early excellence in physical organic chemistry. Journal of Physical Organic Chemistry, 25(8), 611.

[mbo31100-bib-0035] Miller, M. , Zhu, C. , & Bromberg, Y. (2017). clubber: Removing the bioinformatics bottleneck in big data analyses. Journal of Integrative Bioinformatics, 14(2). 10.1515/jib-2017-0020. [Epub ahead of print]. PMC5929469 28609295

[mbo31100-bib-0036] Nikrad, M. P. , Kerkhof, L. J. , & Haggblom, M. M. (2016). The subzero microbiome: Microbial activity in frozen and thawing soils. FEMS Microbiology Ecology, 92(6), fiw081. 27106051 10.1093/femsec/fiw081

[mbo31100-bib-0037] O'Connor, K. , Duetz, W. , Wind, B. , & Dobson, A. D. (1996). The effect of nutrient limitation on styrene metabolism in *Pseudomonas putida* CA‐3. Applied and Environmental Microbiology, 62(10), 3594–3599. 8967774 10.1128/aem.62.10.3594-3599.1996 PMC168165

[mbo31100-bib-0038] Price, P. B. , & Sowers, T. (2004). Temperature dependence of metabolic rates for microbial growth, maintenance, and survival. Proceedings of the National Academy of Sciences of the United States of America, 101(13), 4631–4636. 15070769 10.1073/pnas.0400522101 PMC384798

[mbo31100-bib-0039] Qin, J. , Li, Y. , Cai, Z. , Li, S. , Zhu, J. , Zhang, F. , … Wang, J. (2012). A metagenome‐wide association study of gut microbiota in type 2 diabetes. Nature, 490(7418), 55–60. 23023125 10.1038/nature11450

[mbo31100-bib-0040] Rodriguez‐R, L. M. , Overholt, W. A. , Hagan, C. , Huettel, M. , Kostka, J. E. , & Konstantinidis, K. T. (2015). Microbial community successional patterns in beach sands impacted by the Deepwater Horizon oil spill. The ISME Journal, 9(9), 1928–1940. 25689026 10.1038/ismej.2015.5 PMC4542042

[mbo31100-bib-0041] Rothschild, L. J. , & Mancinelli, R. L. (2001). Life in extreme environments. Nature, 409, 1092. 11234023 10.1038/35059215

[mbo31100-bib-0042] Rusch, D. B. , Halpern, L. A. , Sutton, G. , Heidelberg, K. B. , Williamson, S. , Yooseph, S. , … Craig Venter, J. (2007). The Sorcerer II global ocean sampling expedition: Northwest Atlantic through eastern tropical Pacific. PLOS Biology, 5(3), e77. 17355176 10.1371/journal.pbio.0050077 PMC1821060

[mbo31100-bib-0043] Schloss, P. D. , Gevers, D. , & Westcott, S. L. (2011). Reducing the effects of PCR amplification and sequencing artifacts on 16S rRNA‐based studies. PLoS One, 6(12), e27310. 22194782 10.1371/journal.pone.0027310 PMC3237409

[mbo31100-bib-0044] Schloss, P. D. , Westcott, S. L. , Ryabin, T. , Hall, J. R. , Hartmann, M. , Hollister, E. B. , … Weber, C. F. (2009). Introducing mothur: Open‐Source, platform‐independent, community‐supported software for describing and comparing microbial communities. Applied and Environmental Microbiology, 75(23), 7537. 19801464 10.1128/AEM.01541-09 PMC2786419

[mbo31100-bib-0045] Segawa, T. , Miyamoto, K. , Ushida, K. , Agata, K. , Okada, N. , & Kohshima, S. (2005). Seasonal change in bacterial flora and biomass in mountain snow from the Tateyama Mountains, Japan, analyzed by 16S rRNA gene sequencing and real‐time PCR. Applied and Environmental Microbiology, 71(1), 123–130. 15640179 10.1128/AEM.71.1.123-130.2005 PMC544271

[mbo31100-bib-0046] Shi, Y. , Tyson, G. W. , Eppley, J. M. , & DeLong, E. F. (2010). Integrated metatranscriptomic and metagenomic analyses of stratified microbial assemblages in the open ocean. The ISME Journal, 5, 999. 21151004 10.1038/ismej.2010.189 PMC3131857

[mbo31100-bib-0047] Stibal, M. , Šabacká, M. , & Žárský, J. (2012). Biological processes on glacier and ice sheet surfaces. Nature Geoscience, 5, 771.

[mbo31100-bib-0048] Sunagawa, S. , Coelho, L. P. , Chaffron, S. , Roat Kultima, J. , Labadie, K. , Salazar, G. , … Bork, P. (2015). Ocean plankton. Structure and function of the global ocean microbiome. Science, 348(6237), 1261359. 25999513 10.1126/science.1261359

[mbo31100-bib-0049] Uetake, J. , Tanaka, S. , Segawa, T. , Takeuchi, N. , Nagatsuka, N. , Motoyama, H. , & Aoki, T. (2016). Microbial community variation in cryoconite granules on Qaanaaq Glacier, NW Greenland. FEMS Microbiology Ecology, 92(9), fiw127. 27306554 10.1093/femsec/fiw127

[mbo31100-bib-0050] Vandenbergh, P. A. , & Wright, A. M. (1983). Plasmid involvement in acyclic isoprenoid metabolism by *Pseudomonas putida* . Applied and Environmental Microbiology, 45(6), 1953–1955. 16346325 10.1128/aem.45.6.1953-1955.1983 PMC242567

[mbo31100-bib-0051] Vogel, T. M. , Simonet, P. , Jansson, J. K. , Hirsch, P. R. , Tiedje, J. M. , van Elsas, J. D. , … Philippot, L. (2009). TerraGenome: A consortium for the sequencing of a soil metagenome. Nature Reviews Microbiology, 7, 252.

[mbo31100-bib-0052] Ward, P. G. , Goff, M. , Donner, M. , Kaminsky, W. , & O'Connor, K. E. (2006). A two step chemo‐biotechnological conversion of polystyrene to a biodegradable thermoplastic. Environmental Science & Technology, 40(7), 2433–2437. 16649270 10.1021/es0517668

[mbo31100-bib-0053] Webster‐Brown, J. G. , Hawes, I. , Jungblut, A. D. , Wood, S. A. , & Christenson, H. K. (2015). The effects of entombment on water chemistry and bacterial assemblages in closed cryoconite holes on Antarctic glaciers. FEMS Microbiology Ecology, 91(12), fiv144. 26572547 10.1093/femsec/fiv144

[mbo31100-bib-0054] Wunderlin, T. , Ferrari, B. , & Power, M. (2016). Global and local‐scale variation in bacterial community structure of snow from the Swiss and Australian Alps. FEMS Microbiology Ecology, 92(9), fiw132. 27297721 10.1093/femsec/fiw132

[mbo31100-bib-0055] Yu, K. , & Zhang, T. (2012). Metagenomic and metatranscriptomic analysis of microbial community structure and gene expression of activated Sludge. PLoS One, 7(5), e38183. 22666477 10.1371/journal.pone.0038183 PMC3364235

[mbo31100-bib-0056] Zhang, S. , Yang, G. , Wang, Y. , & Hou, S. (2010). Abundance and community of snow bacteria from three glaciers in the Tibetan Plateau. Journal of Environmental Sciences, 22(9), 1418–1424. 10.1016/s1001-0742(09)60269-2 21174974

[mbo31100-bib-0057] Zhu, C. , Delmont, T. O. , Vogel, T. M. , & Bromberg, Y. (2015). Functional basis of microorganism classification. PLOS Computational Biology, 11(8), e1004472. 26317871 10.1371/journal.pcbi.1004472 PMC4552647

[mbo31100-bib-0058] Zhu, C. , Mahlich, Y. , Miller, M. , & Bromberg, Y. (2018). fusionDB: Assessing microbial diversity and environmental preferences via functional similarity networks. Nucleic Acids Research, 46(D1), D535–D541. 10.1093/nar/gkx1060 29112720 PMC5753390

[mbo31100-bib-0059] Zhu, C. , Miller, M. , Marpaka, S. , Vaysberg, P. , Rühlemann, M. C. , Wu, G. , … Bromberg, Y. (2018). Functional sequencing read annotation for high precision microbiome analysis. Nucleic Acids Research, 46(4), e23. 29194524 10.1093/nar/gkx1209 PMC5829635

